# Uncertainty-Aware Contamination Detection in IoT Water Networks via Interval Type-2 Fuzzy Rare Itemset Mining

**DOI:** 10.3390/s26185974

**Published:** 2026-09-21

**Authors:** Jeya Sutha Mariadhason, Emerson Raja Joseph, Purushothaman Srinivasan, Ramesh Dhanaseelan Francis

**Affiliations:** 1 Department of Computer Applications, St. Xavier’s Catholic College of Engineering, Chunkankadai, Nagercoil 629003, Tamil Nadu, India; jeyasutha@sxcce.edu.in (J.S.M.); dhanaseelan@sxcce.edu.in (R.D.F.); 2 Faculty of Engineering and Technology, Multimedia University, Melaka 75450, Malaysia; m.jeyasutha@mmu.edu.my; 3 Centre for Advanced Analytics, COE of Artificial Intelligence, Faculty of Engineering and Technology, Multimedia University, Melaka 75450, Malaysia; 4 School of Engineering and Technology, Jaipur National University, Jagatpura, Jaipur 302017, Rajasthan, India; dr.s.purushothamann@gmail.com

**Keywords:** anomaly detection, water quality monitoring, sensor data analytics, interval type-2 fuzzy sets, uncertainty modeling, sensor drift, data mining

## Abstract

Uncertainty in low-cost Internet of Things (IoT) sensors challenges real-time contamination identification in institutional water infrastructure. Typically, conventional threshold-based alert systems fail to detect anomalies across multiple correlated parameters that are not individually outside the ‘safe’ limits. We introduce T2MFRM (Type-2 Multiple Fuzzy Rare Itemset Mining), a framework that identifies unusual but significant contamination patterns using uncertainty mining. The system uses Interval Type-2 Fuzzy Sets (IT2FS) to capture sensor measurements under uncertainty by defining a Footprint of Uncertainty (FOU). To mine unusual, rare patterns, we use a hash-table structure with upper-bound pruning to handle the combinatorial explosion associated with mining rare patterns. This preserves computational efficiency for deployment on edge gateways. T2MFRM was benchmarked against FRI-Miner and RP-Growth on six publicly available datasets and ran significantly faster while consuming less memory. The framework’s advantage increased as the maximum frequent-support threshold (minFS) grew from 20% to 36%. We also tested the framework on real-world datasets. One case study demonstrates that, contrary to systems which rely on single-parameter thresholds, T2MFRM detected a multivariate contamination signature in the form of a simultaneous excursion of pH and temperature, neither of which was individually outside of safe limits. The results showed that the proposed system is computationally efficient and increases sensitivity to pollution events in a water management system compared with the traditional Boolean logic approach.

## 1. Introduction

A major public health concern is establishing affordable, safe water monitoring systems for large-scale infrastructure such as hospitals, schools, and industrial complexes. Regular, laboratory-based testing is expensive, time-consuming, and ineffective for monitoring water quality, especially in situations that require rapid monitoring of water contamination. Such systems are insufficient to support health and safety in smart cities [1,2].

IoT-based real-time water quality monitoring using optical and electrochemical sensors has been developed for pH, TDS, turbidity, and temperature measurements [3,4]. One autonomous and networked system of this kind is HydroSense AI, designed for use in institutional settings. Systems such as HydroSense AI have made great progress in developing autonomous, connected hardware; however, all such systems suffer from a common analytical logic issue: heavy reliance on sharp, single-parameter thresholds. Traditional systems can trigger an alert for an event of interest (e.g., pH > 8.5) only when the sharp threshold is crossed. This “Boolean” logic also fails to account for the multi-parametric nature of water quality and contamination, where combinations of multiple parameters near a threshold value may indicate high risk.

In addition to that, inexpensive IoT sensors demonstrate what is called “secondary uncertainty”—an inherent problem of environmental sensing due to electrochemical drift, transducer noise, and biofouling [5,6]. For example, in real-world applications, a pH level of 8.49 may indicate an alkaline spike that exceeds the safe limit of 8.5, but sensor inaccuracies can hide it. Type-1 fuzzy logic is insufficient for such data because it represents uncertainty with a single membership function. Interval Type-2 Fuzzy Logic (IT2FL) [7] is a much better solution because it represents data using linguistic variables with a Footprint of uncertainty (FOU), allowing for reasoning over data while accounting for inaccuracies. Although sensor accuracy and pattern mining in sensor networks are well studied [8,9], contamination detection is essentially a rare-pattern mining task. Namely, the most common “normal” patterns in parameter states in the network are not important; the infrequent ones are, as they indicate water pollution. All rare-pattern solutions, such as RP-Growth [10] or Type-1 fuzzy logic-based FRI-Miner [11], either do not consider sensor uncertainties or consider them inadequately.

To fill this research gap, we introduce the T2MFRM (Type-2 Multiple Fuzzy Rare Itemset Mining) approach. In particular, the algorithm uses IT2FL representation as part of the rare itemset mining process to increase detection sensitivity in the presence of noise. In addition, by using a nested hash table and an upper-bound-based pruning technique, T2MFRM avoids the combinatorial explosion typically associated with rare-itemset mining due to low-support values, enabling edge-gateway implementation.

Main contributions of our work are described as follows:

- **Uncertainty-aware Detection Approach:** An interval type-2 fuzzy rare-itemset mining technique that takes advantage of Interval Type-2 Fuzzy Sets to represent uncertainty due to transducer noise and electrochemical drift within the framework of a Footprint of Uncertainty (FOU);
- **Computationally Efficient Algorithm:** A nested hash-table-based algorithm for rare itemset mining together with Remaining Fuzzy Value (RFV) pruning that has O(1) time access compared to tree-based approaches;
- **Experimental Evaluation on Diverse Datasets:** The T2MFRM algorithm is evaluated on six public datasets against FRI-Miner and RP-Growth algorithms regarding the efficiency of computation as support value gets smaller;
- **Real-world Evaluation using HydroSense AI:** Application of the T2MFRM algorithm on real telemetry data from a campus water network where it identifies a multivariate “pH–temperature” contamination signal which a conventional threshold-based system cannot detect.

## 2. Related Work

Water quality monitoring has evolved from traditional laboratory methods to advanced intelligent IoT solutions. This section reviews the state of the art in three areas: institutional monitoring architectures, uncertainty modeling of sensor measurements, and rare-pattern extraction.

### 2.1. IoT-Based Institutional Water Monitoring

Current research and trends in institutional water quality monitoring focus on “Connected Intelligence,” where the sensing, communication, and application layers are separated for better scalability [1,12]. Example solutions include proposals by [2,12,13], which show the advantage of multi-parameter nodes in real-time monitoring. However, it is noted that advances in affordable sensors for water quality monitoring are a double-edged sword [3]. On one hand, they enable denser deployment; on the other, they increase noise and sensor calibration drift. Existing water quality monitoring platforms, including the validated solution HydroSense AI [14], rely mostly on Boolean logic or LSTM networks to make predictions. This works well for trend forecasting but is not suitable for interpreting “borderline” co-occurrence across several parameters, which could represent a rare contamination signature.

### 2.2. Fuzzy Logic for Sensor Uncertainty

Fuzzy set theory bridges the gap between noisy numeric sensor data and human-like linguistic reasoning [15]. Early fuzzy-set implementations relied on Type-1 Fuzzy Sets to classify sensor readings such as “Low,” “Optimal,” or “High” [16,17]. However, Type-1 Fuzzy Sets rely on a single crisp membership value, whereas environmental sensors have secondary uncertainties—the membership functions drift because of sensor ageing and biofouling [5,7].

IT2FL resolves this problem by including a Footprint of Uncertainty (FOU) and allowing the creation of an interval with tighter bounds for electrochemical transducer tolerances [6,18]. Although IT2FL has been successfully applied to clustering and time-series forecasting in wireless sensor networks [19,20], its use in anomaly detection using data mining remains underexplored.

### 2.3. Frequent vs. Rare Itemset Mining in IoT

Frequent Itemset Mining (FIM) has been the focus of data mining in sensor networks using algorithms such as Apriori [8] or FP-Growth [9]. A comprehensive survey of this area is provided in [21]. But in the context of water safety, discovering frequent patterns provides little information, since they represent the system’s normal condition. The essential information to obtain is contamination, which occurs infrequently.

To discover infrequent but correlated item patterns, Rare Itemset Mining (RIM) has been proposed [22,23]. Related contributions include the minimal rare-itemset generation approach [24]. Examples of RIM algorithms include those using tree data structures, such as RP-Growth [10]. A fast algorithm for mining rare itemsets is proposed in [25]. More recent algorithms incorporate Type-1 fuzzy logic in RIM to deal with quantitative data, e.g., FRI-Miner [11]. Privacy-preserving variants of rare itemset mining have also been explored [26]. However, these approaches remain “uncertainty-blind” or limited to Type-1 fuzzy representation, and they miss borderline rare items or produce many false positives under considerable sensor noise.

Another research avenue applies multivariate deep learning techniques to raw sensor streams to detect anomalies [27], completely bypassing the need for fuzzy representation. These models can serve as classifiers but usually require large amounts of annotated training data and non-interpretable output. In contrast, T2MFRM discovers rare patterns as explicit, human-readable linguistic itemsets (such as “pH = High ∧ Temp= High”), which facility managers can review directly.

### 2.4. Research Gap and Motivation

As illustrated in Table 1, a significant gap remains in frameworks that combine Type-2 uncertainty modeling and Rare Itemset Mining. Currently available RIM algorithms (such as RP-Growth) work on crisp data and are therefore prone to malfunctioning due to sensor drift. At the same time, currently available fuzzy mining algorithms (such as FRI-Miner) use Type-1 fuzzy sets, which cannot model fluctuations in IoT sensor accuracy.

## 3. Preliminaries and Problem Formulation

This section covers the mathematical concepts behind representing sensor uncertainty using Interval Type-2 Fuzzy Sets (IT2FS) and rare-pattern mining metrics.

### 3.1. Notation and Data Representation

Let $I=\{i_{1},i_{2},\ldots ,i_{m}\}$ be a set of *m* distinct sensor parameters (e.g., pH, TDS, and Turbidity). A quantitative transaction database $D=\{T_{1},T_{2},\ldots ,T_{n}\}$ consists of *n* discrete sampling events. Each transaction $T_{q}$ is uniquely identified by a timestamp $T_{id}$ and contains numerical readings $v_{i,q}$ for each sensor.

**Definition 1**  (Linguistic *k*-itemset)**.** *A k-itemset is a set of k distinct linguistic terms (e.g., pH=High, Temp=High). An itemset is considered a subset of a transaction $T_{q}$ if the fuzzy membership values of its constituent terms are non-zero*.

### 3.2. Uncertainty Modeling via Interval Type-2 Fuzzy Sets

**Figure 1:**
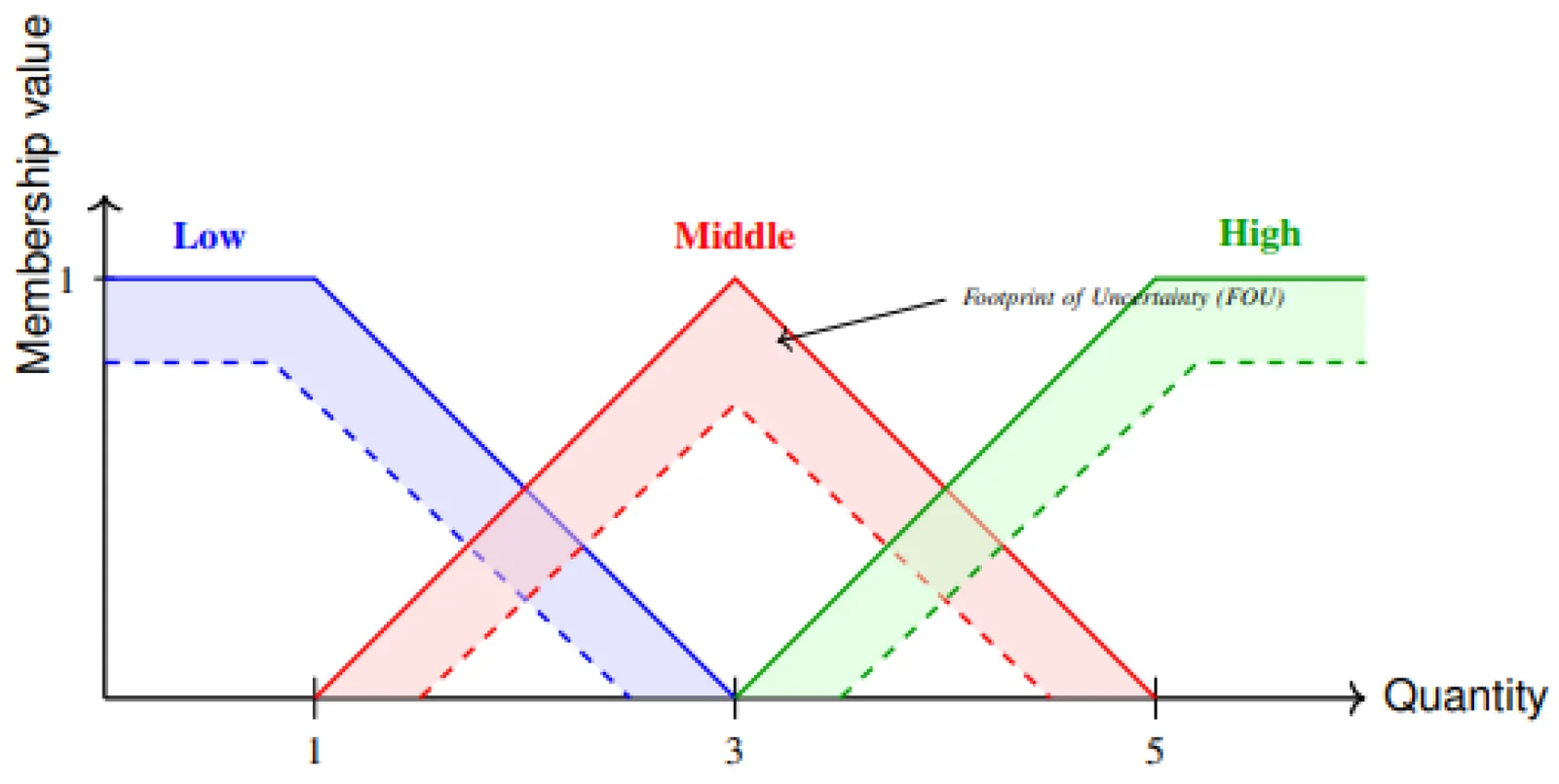
Interval Type-2 Fuzzy membership functions (Low, Middle, and High) used for sensor data fuzzification. The shaded regions represent the Footprint of Uncertainty (FOU), capturing transducer noise and drift.

To account for sensor noise and electrochemical drift, every raw reading $v_{i,q}$ is mapped to a set of *h* linguistic terms $\left\{R_{i1},R_{i2},\ldots ,R_{ih}\right\}$ (typically Low, Middle, and High). Unlike traditional fuzzy logic, we use Interval Type-2 Fuzzy Sets (IT2FS) to represent the Footprint of Uncertainty (FOU) (Figure 1).

**Definition 2**  (Interval Membership)**.** *The membership of a sensor reading $v_{i,q}$ in a linguistic term $R_{ij}$ is defined as a closed interval:*

(1) $$f_{i,q}=\left[fv_{ij,q}^{lower},\;fv_{ij,q}^{upper}\right]$$

where $fv^{lower}$ and $fv^{upper}$ represent the Lower Membership Function (LMF) and Upper Membership Function (UMF), respectively. This interval captures the “secondary uncertainty” of the IoT probe, ensuring that minor fluctuations in voltage do not lead to a total loss of membership.

A terminological clarification is needed here, since the two are often conflated. Noise, in this work, refers to short-term, zero-mean stochastic fluctuations in a sensor’s raw output (e.g., transient electrical interference), which conventional signal conditioning or averaging can largely suppress. Measurement uncertainty, by contrast, is the bounded but not fully eliminable ambiguity that remains about a sensor’s true reading after such conditioning, arising from systematic effects such as electrochemical drift, calibration ageing and biofouling. The IT2FS/FOU representation adopted here is designed to model the latter: the width of the FOU reflects the plausible discrepancy between an observed reading and the reading a freshly calibrated, unfouled probe would report, not the instantaneous jitter that onboard averaging already removes. Where the remainder of this manuscript refers loosely to sensor “noise,” it denotes this systematic residual uncertainty unless stated otherwise.

**Definition 3**  (Type Reduction)**.** *To ensure computational tractability during support counting, a centroid-based reduction is applied to produce a reduced crisp fuzzy value ($fv_{ij,q}^{c}$):*

(2) $$fv_{ij,q}^{c}=\frac{fv_{ij,q}^{lower}+fv_{ij,q}^{upper}}{2}$$

This reduced value represents the “center of gravity” of the sensor’s uncertainty for that specific reading.

### 3.3. Support Metrics and Rareness Constraints

The T2MFRM algorithm utilizes a nested hash-table structure to manage data efficiently. Two key metrics govern the mining process:

**Definition 4**  (Current Fuzzy Value, CFV)**.** *For a multivariate linguistic term (itemset) X in transaction $T_{q}$, the CFV is calculated using the minimum t-norm operator:*

(3) $$CFV\left(X,T_{q}\right)=\min\left\{fv_{i,j,q}^{c}|\left(i,j\right)\in X\right\}$$

**Definition 5**  (Remaining Fuzzy Value, RFV)**.** *The RFV serves as an upper-bound pruning heuristic. It represents the maximum possible fuzzy support that can be gained by extending the current itemset with subsequent terms in the predefined processing order. This allows the algorithm to prune “hopeless” branches of the search tree early, saving memory*.

### 3.4. Problem Statement

The objective of T2MFRM is to extract the complete set of Multiple Fuzzy Rare Itemsets (MFRIs). To distinguish genuinely rare contamination patterns from either frequent “normal” states or insignificant sensor noise, we apply two user-defined thresholds:

1. Minimum Rare Support (minRS): The floor threshold that filters out insignificant noise.
2. Maximum Frequent Support (minFS): The ceiling threshold that excludes common, healthy water states.

An itemset *X* is identified as an MFRI if its cumulative support S(X) satisfies the dual-threshold constraint given by Equation (4):

(4) minRS×|D|≤S(X)≤minFS×|D|

where

(5) $$S\left(X\right)=\sum_{q=1}^{n}CFV\left(X,T_{q}\right).$$

**Definition 6**  (Normal Pattern)**.** *An itemset X is a normal pattern if S(X)>minFS×|D|. Normal patterns describe the system’s typical, healthy operating states and are deliberately excluded from the mining output, since their high incidence carries little diagnostic value for contamination screening*.

**Definition 7**  (Rare Pattern/Candidate Contamination Signature)**.** *An itemset X is a rare pattern—equivalently, a candidate contamination signature—if it satisfies the dual-threshold constraint of Equation (4), i.e., minRS×|D|≤S(X)≤minFS×|D|. Itemsets with S(X)<minRS×|D| are not reported: at the available sample size their incidence is too low to be reliably distinguished from the residual measurement uncertainty discussed in Section 3.2, and reporting them would risk mistaking that uncertainty for a genuine, reproducible pattern*.

## 4. The T2MFRM Algorithm

**Figure 2:**
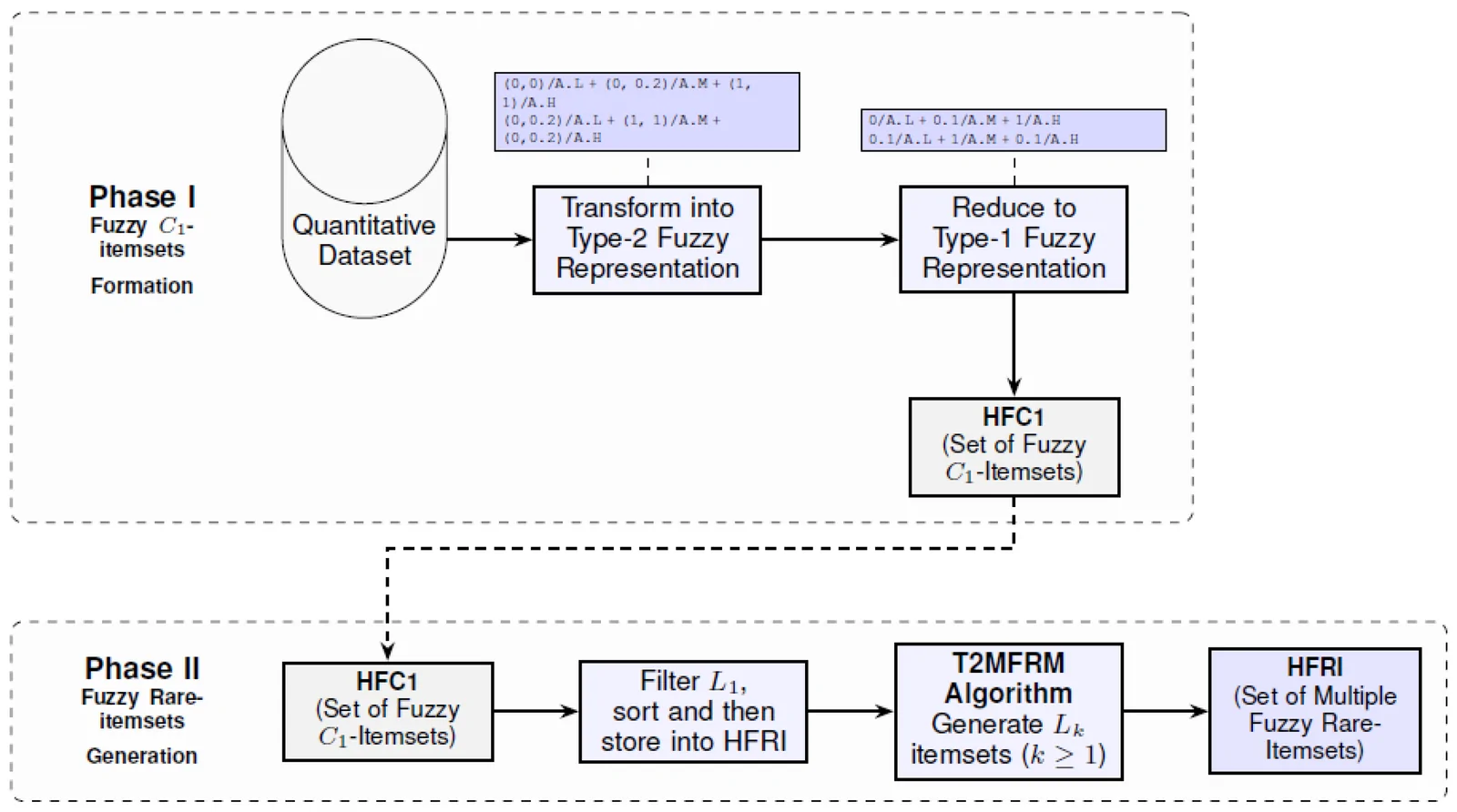
Unified architecture of the T2MFRM framework. Phase I illustrates the transformation of raw sensor telemetry into reduced fuzzy values; Phase II depicts the recursive rare-itemset extraction logic via the nested hash-table structure.

To overcome the computational challenges of mining rare patterns in high-uncertainty numeric data, T2MFRM utilizes a two-phase hierarchical hash-table approach. This architecture ensures efficient storage of linguistic terms and enables O(1) retrieval speeds, which is critical for real-time monitoring. Figure 2 illustrates the overall framework architecture.

### 4.1. Phase I: Fuzzy $C_{1}$-Itemset Formation

In the first phase, the algorithm performs a single scan of the sensor database *D* as summarized in Algorithm 1. For each transaction $T_{q}$ (sampling event), every sensor reading $v_{i,q}$ is processed as follows:

1. **Fuzzification:** Raw numeric values are transformed into Interval Type-2 linguistic terms {Low, Middle, and High} using the Upper and Lower membership functions. This step explicitly captures the sensor’s measurement tolerance.
2. **Type Reduction:** Each interval is reduced to a crisp fuzzy value ($fv^{c}$) using the centroid method to facilitate additive support counting.
3. **Nested Storage:** The resulting values are stored in a nested hash-table structure, HFC1. The primary keys are the sensor items, and the sub-table HFC1S maps transaction IDs ($T_{id}$) to their specific fuzzy memberships.
4. **Incremental Aggregation:** The total support for each linguistic term is updated incrementally. By the end of Phase I, the algorithm has a complete map of how often every sensor state occurred across the entire deployment.

**Algorithm 1.**
(Fuzzy $C_{1}$-Itemset Formation). This algorithm scans the database once to transform raw sensor readings into uncertainty-aware fuzzy representations.**Input:** *D* (Quantitative sensor dataset); μ (Collection of Interval Type-2 membership functions).**Output:** HFC1 (Nested hash table containing fuzzy candidate 1-itemsets and metadata).
1.Initialize HFC1←⌀2.**for each** transaction $T_{q}\in D$ **do**3. **for each** sensor item $i_{j}\in T_{q}$ **do**4.  Retrieve raw numeric value $v_{i_{j},q}$5.  *// Step 1: Fuzzification into IT2FS values*6.  Calculate $\left[fv^{lower},fv^{upper}\right]$ for linguistic regions {Low, Middle, High} using μ7.  *// Step 2: Centroid-based Type Reduction*8.  $fv^{c}\leftarrow \left(fv^{lower}+fv^{upper}\right)/2$9.  **if** $fv^{c}>0$ **then**10.   Store $fv^{c}$ in sub-hashtable HFC1S keyed by $T_{id}$11.   Update cumulative support $S\left(R_{ijl}\right)\leftarrow S\left(R_{ijl}\right)+fv^{c}$12.  **end if**13. **end for**14.**end for**15.**return** HFC1

### 4.2. Phase II: Multiple Fuzzy Rare Itemset Extraction

The second phase identifies rare co-occurrences by recursively exploring combinations of the 1-itemsets generated in Phase I. This phase is governed by the dual-threshold constraint (minRS and minFS).

**Step A: Generation of $L_{1}$-itemsets.** The algorithm filters HFC1 to identify all individual linguistic terms that are “rare”—meaning they occur enough to be significant but not enough to be considered “normal” operation. These terms form the foundation for multivariate analysis.

**Step B: Recursive Generation of $L_{k}$-itemsets (k≥2).** To find co-occurring patterns (e.g., {pH = High ∧ TDS = High}), the algorithm joins overlapping terms. To prevent the “combinatorial explosion” common in data mining, we implement Remaining Fuzzy Value (RFV) pruning:

- For any candidate pattern, the algorithm calculates its RFV—the maximum potential support it could achieve if extended.
- If the RFV is lower than the minRS threshold, the entire branch of the search tree is discarded (pruned).
- This ensures that the system does not waste memory or CPU cycles processing co-occurrences that can never meet the criteria for a contamination signature.

The complete recursive rare-itemset extraction process incorporating this pruning strategy is formalized in Algorithm 2.
**Algorithm 2.**
(Multiple Fuzzy Rare-Itemsets (MFRI) Generation). This algorithm recursively explores the search space and uses the RFV metric to prune insignificant branches.**Input:** HFC1 (from Algorithm 1); minRS (min rare support); minFS (max frequent support).**Output:** HFRI (The set of all detected multivariate rare patterns).
1.*// PART I: Filter for Rare 1-Itemsets ($L_{1}$)*2.$L_{1}\leftarrow \left\{R_{ijl}\in HFC1|minRS\leq S\left(R_{ijl}\right)\leq minFS\right\}$3.Sort $L_{1}$ in ascending order of support4.*// PART II: Heuristic computation of Remaining Fuzzy Value (RFV)*5.**for each** term $X\in L_{1}$ **do**6. Calculate RFV(X) as the upper bound of possible support extensions.7.**end for**8.*// PART III: Recursive generation of $L_{k}$-itemsets (k>1)*9.**function** GenerateMFRI(itemsets, candidates)10. **for each** C∈candidates **do**11.  NewSet←itemset∪C12.  $S\left(NewSet\right)\leftarrow \sum \min\left(fv_{c}^{itemset},fv_{c}^{C}\right)$ *// T-norm intersection*13.  **if** S(NewSet)≥minRS and S(NewSet)≤minFS **then**14.   Add NewSet to HFRI15.  **end if**16.  *// Pruning logic*17.  **if** RFV(NewSet)≥minRS **then**18.   GenerateMFRI(NewSet,remainingcandidates)19.  **end if**20. **end for**21.**end function**

### 4.3. Complexity Analysis

The design of T2MFRM is optimized for the resource constraints of IoT edge gateways:

- **Time Complexity:** Phase I is $O(N\times L_{avg}\times h)$, where *N* is the number of transactions. This is linear and highly efficient for streaming sensor data. In Phase II, the use of the hash-table enables O(1) amortized access time for checking itemset memberships, significantly outperforming the O(logN) or O(depth) speeds of tree-based models like RP-Growth.
- **Space Complexity:** Unlike tree-based algorithms that must store complex pointer structures for every possible path, T2MFRM uses a compact hash-based layout. Its space complexity is $O(m\times h\times N_{avg})$, where $N_{avg}$ is the average number of transactions per term. This results in a “flat” memory footprint that remains stable even as the search space expands, as shown in our experimental results (Section 5.3).

## 5. Algorithm Validation on Public Benchmarks

To evaluate T2MFRM’s computational performance and scalability, we benchmarked it against two established baselines: RP-Growth [10] and FRI-Miner [11]. RP-Growth is a standard, tree-based mining algorithm, and FRI-Miner is a Type-1 mining algorithm that uses fuzzy logic.

### 5.1. Experimental Setup and Dataset Properties

We implemented the algorithms in MATLAB R2026a and ran them on an Intel Core i5-12450H CPU with 32 GB of RAM, running Windows 11. We selected six benchmark datasets representing a spectrum of data characteristics, from small and dense (Breast Cancer) to massive and highly sparse (Kosarak). The dataset properties are summarized in Table 2.

We fuzzified the data values using Interval Type-2 membership functions. To be consistent and fair, we set the breakpoints in the fuzzy region based on the data: [1,5,10] for the Breast Cancer data, and [100,500,900] for the other five datasets.

These six datasets are used exclusively to characterize T2MFRM’s computational scalability—runtime and memory behavior across a controlled range of transaction density, sparsity and dimensionality—which is standard practice in the itemset-mining literature precisely because no public benchmark of comparable scale and diversity exists for water-quality telemetry. This benchmarking makes no claim about domain-specific detection accuracy. Domain-relevant validation—whether T2MFRM’s output is meaningful for real water quality contamination detection—is instead provided separately in Section 6 using real multi-parameter telemetry from an operational deployment, and its sensitivity to methodological choices is further examined in Section 6.6.

### 5.2. Evaluation of Execution Time (Runtime)

**Figure 3:**
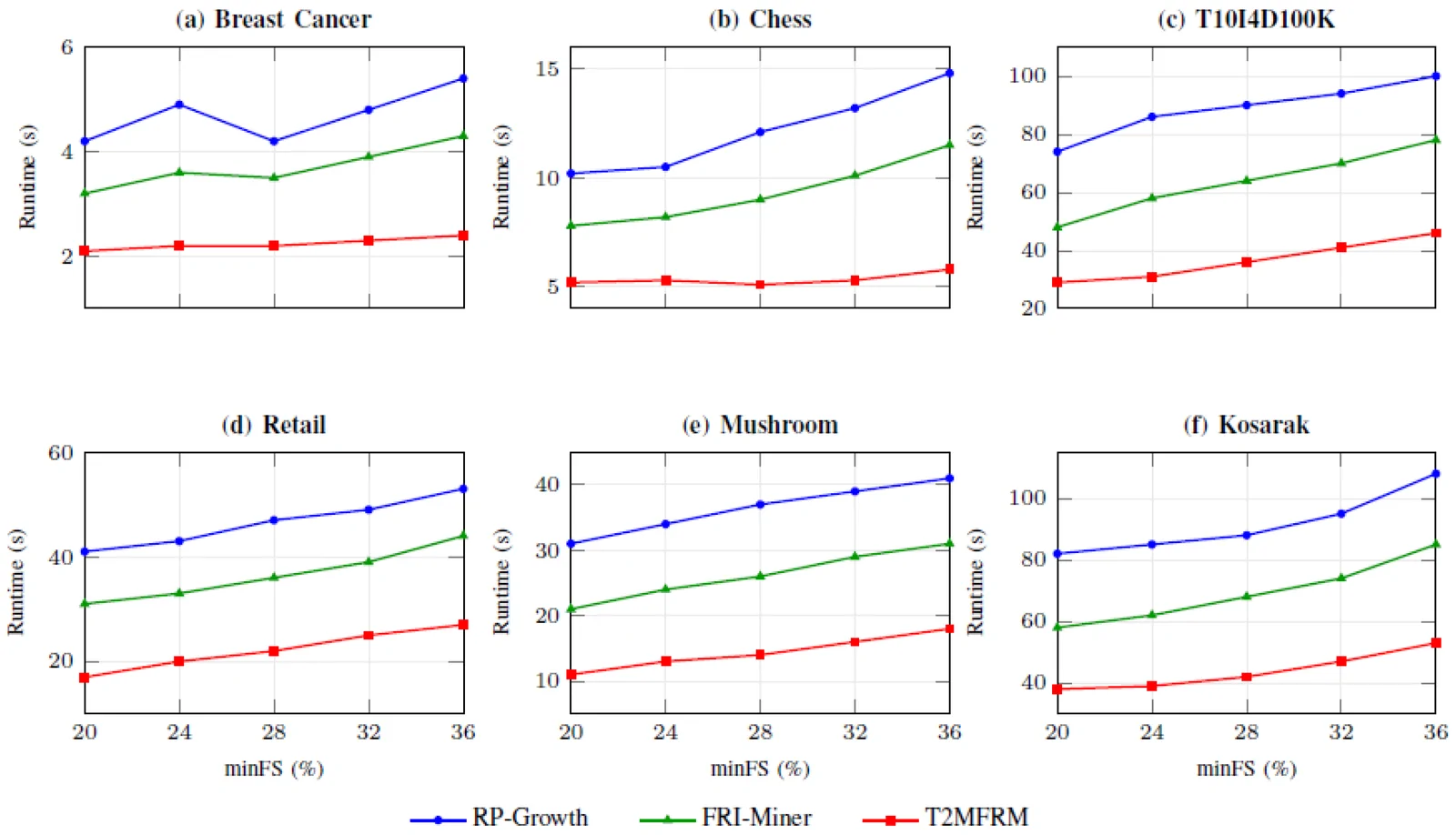
Comparison of execution time (seconds) vs. Maximum Frequent Support (minFS) threshold across six benchmark datasets (**a**–**f**). T2MFRM (red) demonstrates consistently lower runtime compared to RP-Growth and FRI-Miner.

To compute runtime, we set minRS to 10% and varied minFS from 20% to 36%. Figure 3a–f shows that across all benchmark data, T2MFRM achieved the smallest runtime for all threshold values.

The difference in runtime was dramatic. In the Chess data, for example (Figure 3b), while T2MFRM took values of 5–6 s, both RP-Growth and FRI-Miner took values closer to 15 s and 12 s, respectively. Further, T2MFRM’s runtime advantage was even more significant on the larger, sparser datasets (T10I4D100K and Kosarak) at the upper end of the data size in Figure 3c and Figure 3f, respectively, where the tree-based RP-Growth took values greater than 100 s. In comparison, T2MFRM took values less than 55 s.

T2MFRM uses a multi-level hash table. This allows for O(1) access to fuzzy memberships, achieving superior speed over other fuzzy pattern mining frameworks. FRI-Miner and RP-Growth rely on intersecting vertical lists and traversing deep tree structures, respectively, both of which are expensive operations as the number of items increases.

### 5.3. Analysis of Memory Consumption

**Figure 4:**
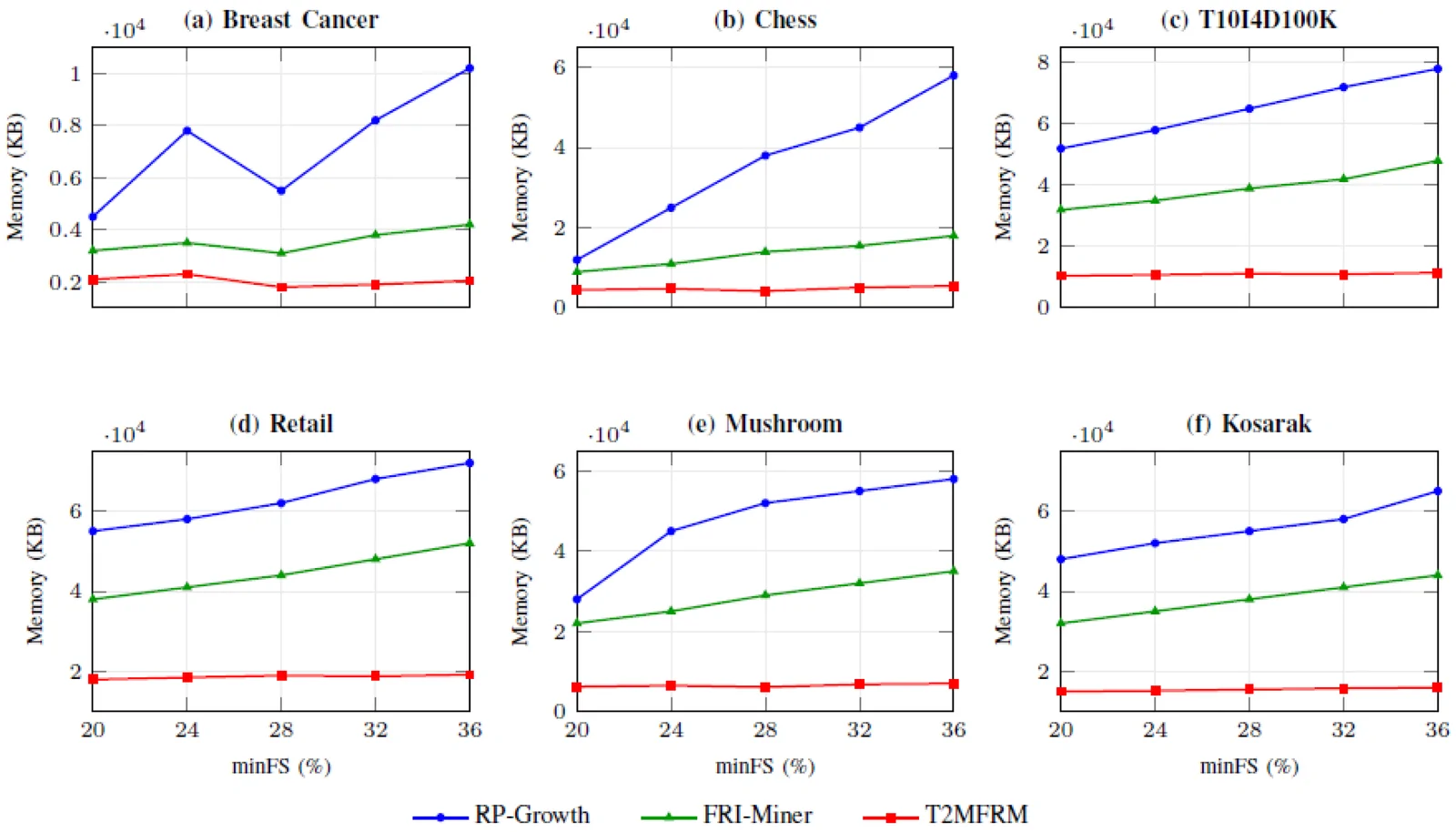
Measured memory usage (KB) across varying minFS thresholds. T2MFRM exhibits a stable, “flat” memory footprint, outperforming tree-based baselines as data sparsity and dimensionality increase.

Memory efficiency is a key requirement in sensor and edge-gateway deployments. Figure 4a–f shows T2MFRM has an extremely “flat” memory footprint. Unlike the baselines, memory usage barely increases as the minFS threshold rises.

For the large, sparse datasets (Figure 4c,f), T2MFRM’s memory usage was nearly constant, while RP-Growth’s increased by a factor of six. Because T2MFRM uses a compact hash structure, memory usage for RP-Growth and other tree-based methods increases dramatically due to an explosion of memory nodes in high-dimensional data.

### 5.4. Pattern Richness and Granularity

**Figure 5:**
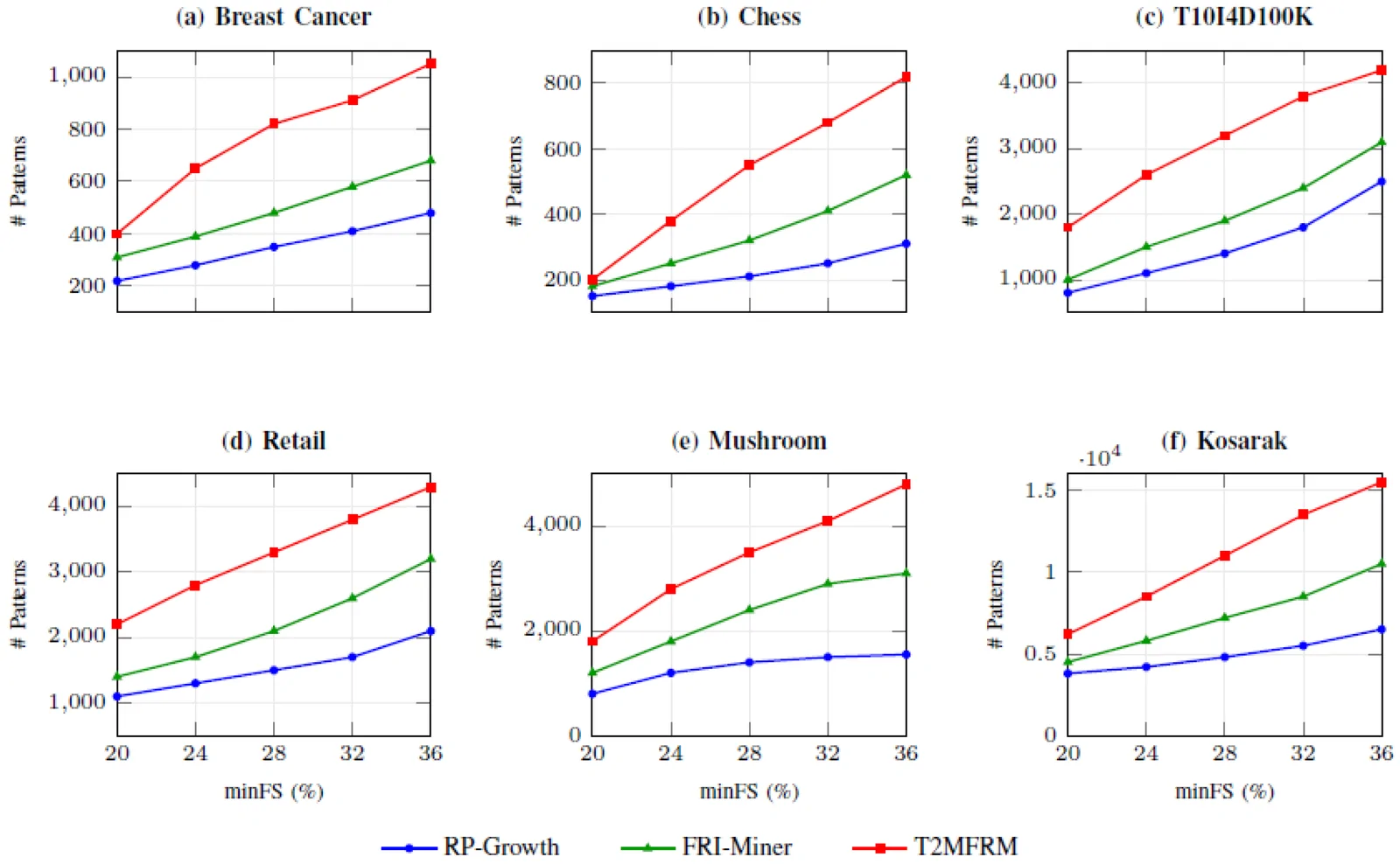
Number of extracted rare patterns vs. minFS threshold. The higher pattern volume identified by T2MFRM reflects its ability to capture overlapping multivariate co-occurrences that are “clipped” by Boolean or Type-1 logic.

In addition to speed and memory, we evaluated the volume of patterns extracted. Figure 5a–f shows that T2MFRM consistently identifies a larger number of patterns than both baselines—typically 1.5 to 2 times more than FRI-Miner.

A higher pattern count initially appears to be “noise,” but in Interval Type-2 Logic, it actually represents a higher degree of detail. Given that T2MFRM addresses overlapping fuzzy regions (a reading that is 40% “Medium” and 60% “High”), it incorporates borderline co-occurrences that both Boolean Logic (RP-Growth) and Type-1 Logic (FRI-Miner) simply “clip.” Increased sensitivity is essential for early-warning systems, where a contamination event begins as a minor, multi-parameter shift.

### 5.5. Discussion of Benchmark Results

The benchmark results show that the technical design of T2MFRM, specifically hash-table retrieval and RFV elimination, reaches practical runtimes. By separating fuzzy representation from search-tree complexity, T2MFRM is a feasible choice for real-time analysis of sensor data on embedded systems with many constraints.

## 6. Field Case Study on HydroSense AI Telemetry

To assess the viability of our proposed technique, we tested it on telemetry from the HydroSense AI Institutional Monitoring Platform. We used real-world telemetry to test whether the rare mining approach, with uncertainty consideration, could produce results that were “invisible” to standard Boolean threshold logic.

### 6.1. Dataset and Fuzzification Parameters

The study utilized a dataset of 644 valid multi-parameter readings spanning 8 April–19 May 2026. Each transaction included pH, turbidity (NTU), temperature (°C), and TDS (ppm). To simulate real-world deployment, the linguistic breakpoints were informed by WHO Drinking-Water Guidelines [28].

- pH: Safe range 6.5–8.5.
- TDS: Limit < 500 ppm.
- Turbidity: Limit < 5 NTU.
- Temperature: A tercile split based on the deployment’s seasonal range.

T2MFRM was executed with minRS=1% (to filter out insignificant outliers) and minFS=15% (to exclude the frequent “normal” co-occurrences of a healthy system).

The width of the Footprint of Uncertainty (FOU) around each crisp breakpoint above is itself a design parameter and is documented explicitly here, since it was previously left implicit. For every parameter, the Upper Membership Function (UMF) spans a symmetric transition band of ±1% of the parameter’s operating range around the relevant WHO-anchored breakpoint, and the Lower Membership Function (LMF) spans half of that band (±0.5%), so that LMF(x)≤UMF(x) everywhere, as required for a valid interval type-2 fuzzy set. This 1%-of-range UMF width is the setting used to generate Table 3 and is treated as the “Medium (baseline)” level in the sensitivity analysis of Section 6.6, which also reports results at half (Narrow, 0.5%) and double (Wide, 2%) this width, together with ±5% and ±10% perturbations of the breakpoints themselves. The type reduction from the resulting [LMF, UMF] interval to the crisp fuzzy value used in scoring is the midpoint average of Equation (2).

### 6.2. Detected Multivariate Rare Patterns

**Figure 6:**
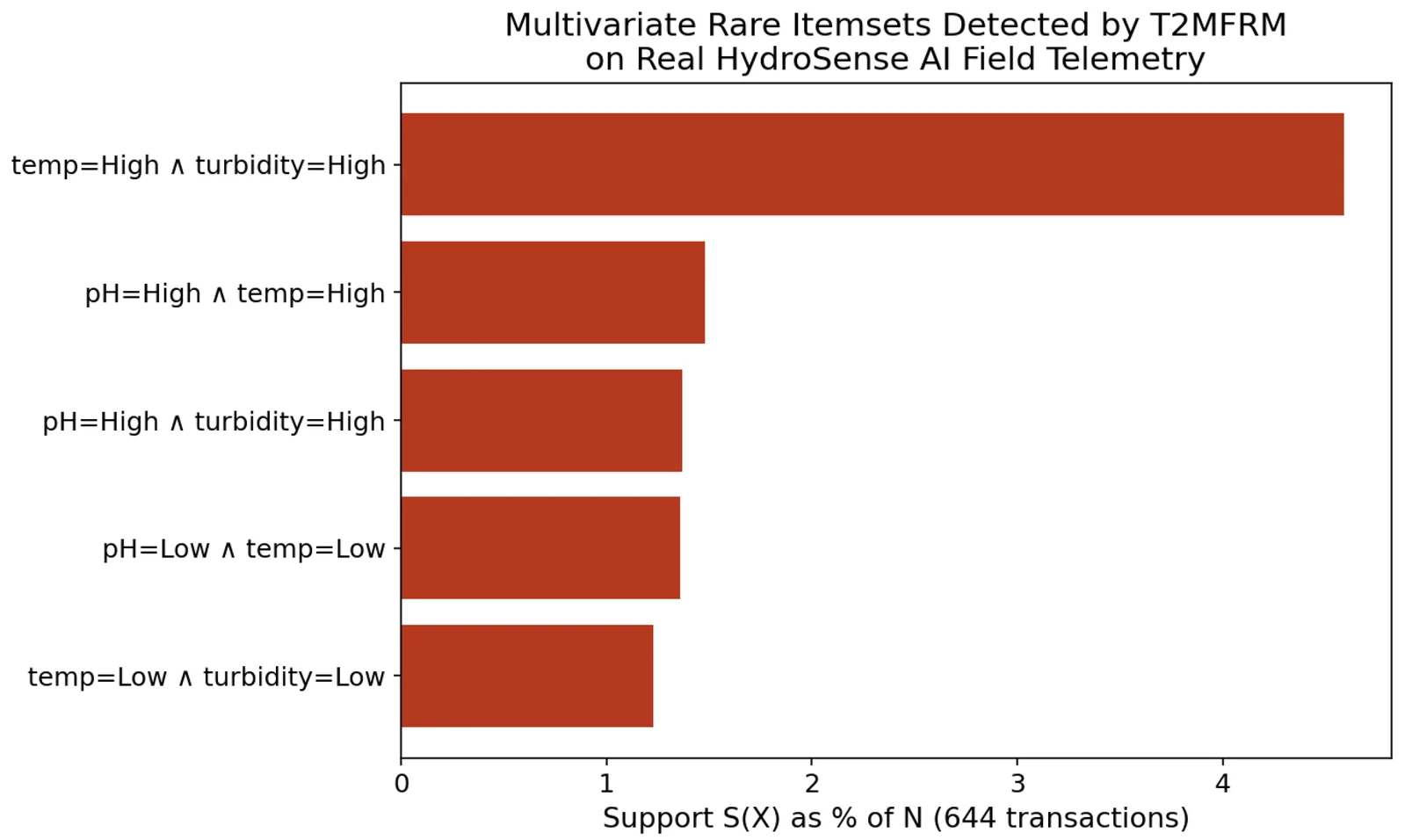
Ranking of multivariate rare itemsets detected in the HydroSense AI campus deployment. The support S(X) is expressed as a percentage of the total transactions (N=644).

The multivariate rare itemsets identified from the HydroSense AI campus telemetry are illustrated in Figure 6, which ranks each candidate contamination pattern according to its cumulative fuzzy support percentage (S(X) as a percentage of N=644) corresponding to the values reported in Table 3.

### 6.3. Flagship Case Study: The pH–Temperature “Borderline” Excursion

The itemset {pH = High AND Temp = High} was the most interesting result. This itemset was present in 80 transactions (approximately 12.4% of the 644 transactions). The cumulative fuzzy support for this itemset was S(X)=9.53 (approximately 1.48% of *N*, see Table 3) and was observed during the second week of April.

**Figure 7:**
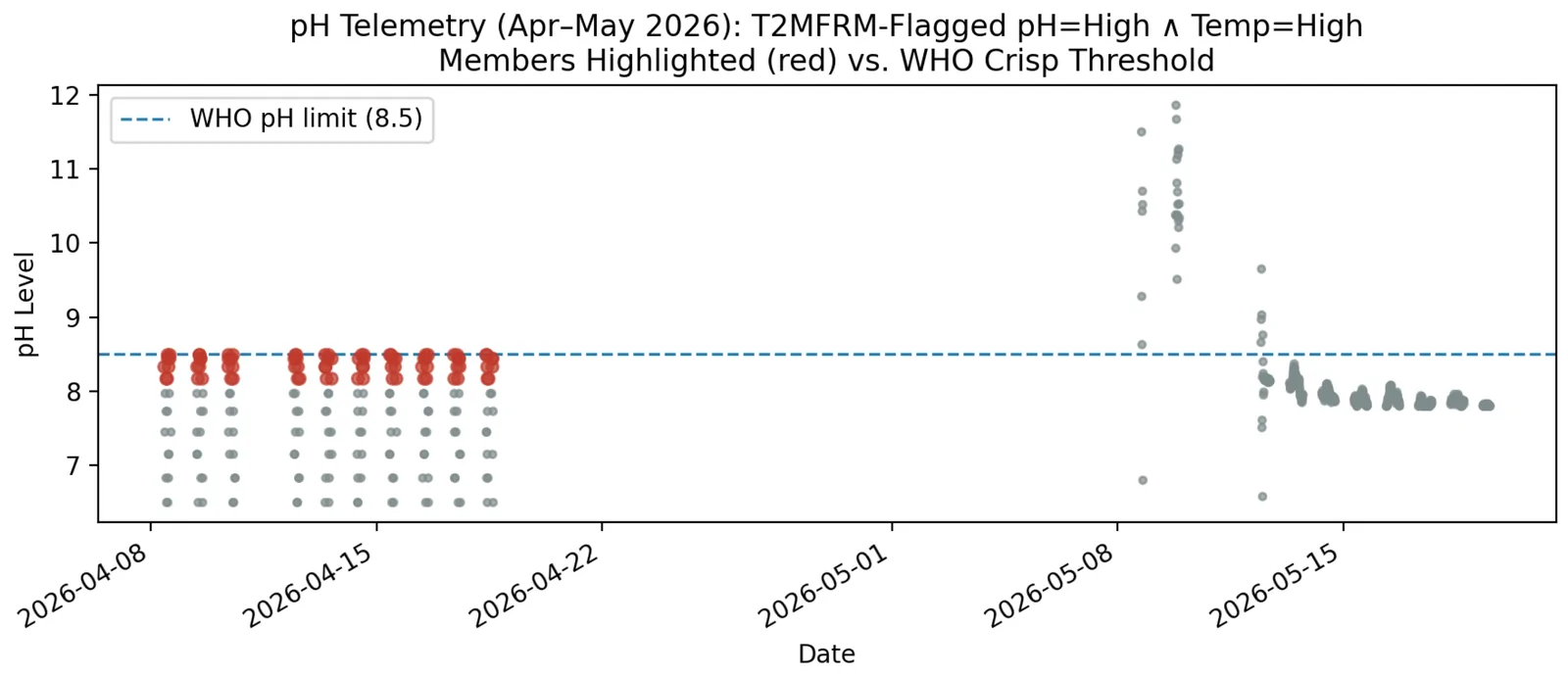
pH telemetry from the April deployment phase. Red markers indicate co-occurrences of {pH = High ∧ Temp = High} identified by T2MFRM. Note that these “near-miss” events remain below the 8.5 WHO safety limit (dashed line) and would be missed by standard thresholding.

In Figure 7 these transactions have an average pH of 8.49. As the original HydroSense AI paper [14] and most water management systems use Boolean logic, these readings would not trigger a water quality breach warning because they are below the 8.5 safety threshold.

However, the fuzzy logic used in T2MFRM classifies these readings as in the ‘High’ category. By showing this marginal pH state coupled with a temperature reading of 35 °C, the algorithm defines a multivariate contamination signature that a univariate system cannot represent. This signaling system allows facility managers to respond a few hours or a day before the system exceeds a regulatory limit.

### 6.4. Distinguishing Contamination from Sensor Faults

The algorithm found a group of pH readings around 11.86 on 8–9 May 2026. The reading co-occurred with near-zero turbidity and TDS.

T2MFRM recognized these patterns as rare; however, we believe they are more attributable to an instrumentation artifact (e.g., sensor maintenance) than to legitimate chemical pollution. Rare Itemset Mining is a useful approach because it helps focus on the important aspects of the problem. It reduces thousands of data points to significant patterns attributable to true pollution rather than sensor artifacts identified by a secondary AI classifier.

### 6.5. Discussion of Field Results

The transition to the proactive mining of rare items replaced the reactive threshold approach and resulted in the following observations:

- **Enhanced Sensitivity:** In addition to threshold violations, the approach captures “near-miss” events that Boolean logic systems ignore.
- **Mitigation of Data Fatigue:** It eliminates the frequent “normal” data streams that facility managers are not interested in.
- **Capture of Complex Impacts:** Many water chemistry inter-relationships (as in the case of pH and Temp) become discernible only in the context of a unique water source.

### 6.6. Sensitivity and Robustness Analysis

This section reports a dedicated study addressing both, conducted on the same HydroSense AI telemetry used in Section 6.1, Section 6.2, Section 6.3, Section 6.4 and Section 6.5 (638 valid transactions after removing 20 idle-sensor sentinel readings), using a fuzzification and itemset-scoring pipeline re-implemented in MATLAB R2026a strictly from the definitions in Section 3. Because the original benchmark implementation used to generate Table 3 was not preserved as an independently re-runnable artifact, this re-implementation reproduces Table 3’s flagship {pH = High AND Temp = High} result closely (Table 4, 0% shift, Medium width row) but not to the last decimal, and should be read as a transparent, independently reproducible companion analysis rather than a bitwise reproduction of Table 3. The fully documented MATLAB code, the prepared telemetry extract, and a flow diagram of the analysis pipeline are provided as Supplementary Materials.

**Sensitivity to Membership-Function Breakpoints and FOU Width.** Table 4 reports the support and detection status of the flagship {pH = High AND Temp = High} itemset, as the WHO-anchored breakpoints (pH, TDS, turbidity, and the two temperature tercile cut-points) are jointly shifted by −10%, −5%, 0% (baseline), +5%, and +10%, at each of the three FOU-width levels defined in Section 6.1 (Narrow 0.5%, Medium 1.0%, and Wide 2.0%), holding minRS=1% and minFS=15% fixed at their Section 6.1 values.

At the baseline (0%) breakpoints, the flagship pattern is recovered at all three FOU widths—support rises from 6.47 (1.01% of *N*) at Narrow width to 14.25 (2.23% of *N*) at Wide width—so FOU width alone, over the 0.5–2% range tested, does not change the qualitative outcome. Breakpoint placement is far more consequential: shifting the breakpoints down by 5% or 10% moves the pattern’s support up sharply (25.9–89.2% of *N*, at Medium width), pushing it past the minFS=15% ceiling so it is reclassified as a normal rather than a rare state; shifting the breakpoints up by 5% or 10% eliminates the pattern entirely (support =0%), because no reading in the April deployment window reaches the raised threshold. This directly confirms that the ‘9.53/1.48% of *N*’ figure reported in Table 3 is a property of the specific WHO-anchored breakpoints used, not a deployment-independent constant, and a ±5% breakpoint miscalibration is sufficient to make the flagship pattern either vanish or blend into the normal baseline.

Table 5 additionally performs the joint minRS/minFS perturbation, holding breakpoints at baseline and varying the dual-threshold pair alongside the three FOU widths.

The flagship pattern is lost only under the tightest threshold pair tested (minRS=2%, minFS=20%) at Narrow and Medium FOU width, where its support (6.47–9.71) falls below the raised minRS cut-off (12.76); it is retained at Wide width and under both looser threshold pairs. Threshold choice therefore compounds, rather than replaces, the breakpoint-placement sensitivity documented above.

**Computational Efficiency Under Sensor Drift and Edge Cases.** Computational efficiency is preserved when the input degrades—i.e., under sensor drift or edge-case conditions—rather than only under the clean, high-quality benchmark data of Section 5. We timed the same re-implemented pipeline (MATLAB R2026a) on four conditions built from the same telemetry: (A) the full clean deployment; (B) a normal-only subset excluding the real 8–9 May sensor-fault window already discussed in Section 6.4; (C) that real fault window in isolation; and (D) the full deployment with additional synthetic electrochemical drift and Gaussian jitter injected on top of the real signal, to emulate accelerated sensor ageing beyond what occurred in the field. The computational performance, runtime, and memory metrics across these four scenarios are summarized in Table 6.

Throughput remains essentially flat (3.4–3.9 transactions/ms) and the number of detected itemsets stays within a similarly narrow range (3–7) across all four conditions: neither the per-transaction computational cost nor the output volume diverges when the input is degraded by a real instrumentation fault or by injected drift, consistent with the O(1) hash-table access argument of Section 4. As with the Section 5 benchmarks, absolute millisecond and kilobyte values are specific to this single-machine MATLAB implementation and will vary by hardware; the flat, non-diverging pattern across conditions—rather than the absolute numbers—is the finding being reported here.

## 7. Conclusions, Limitations, and Future Work

### 7.1. Conclusions

This paper introduced T2MFRM (Type-2 Multiple Fuzzy Rare Itemset Mining), an uncertainty-aware algorithm designed to enhance contamination early-warning in institutional water networks. By integrating Interval Type-2 Fuzzy Logic directly into the rare-pattern mining process, the framework explicitly models the inherent noise and electrochemical drift of low-cost IoT sensors within a Footprint of Uncertainty (FOU).

Performance assessments across multiple dataset types confirmed that T2MFRM’s nested hash table design and Remaining Fuzzy Value (RFV) pruning technique deliver significant gains over both traditional tree-based algorithms and Type-1 fuzzy techniques. T2MFRM retains a stable, “flat” memory footprint and performs faster, making it a good choice for deployment on resource-constrained edge computing devices.

Validation using field telemetry collections from the HydroSense AI system confirmed the approach’s utility. T2MFRM detected a signature of multivariate contamination. It was an excursion of elevated pH and temperature combined. Of significance is that this signature, when viewed by conventional means, would technically fall within “safe” limits and therefore was “invisible” to Boolean threshold-based algorithmic techniques. From this work, it is clear that T2MFRM has the potential to be a superior tool for the “triage” of large volumes of data to summarize the results to a manageable number of high-priority diagnostically significant patterns.

### 7.2. Limitations

The present study has several limitations that should be considered alongside its contributions. First, the membership function design is fixed during the initial construction stages, and fuzzy representations will likely remain sensitive to these designs. Although we set breakpoints based on the WHO, site-specific calibration is still needed—Section 6.6 quantifies this dependency directly, showing that shifting the WHO-anchored breakpoints by as little as ±5% moves the flagship pH–temperature pattern’s support either above the maximum-frequent-support ceiling (reclassifying it as a normal state) or to zero (eliminating it outright), depending on the direction of the shift. The absolute support values in Table 3 should therefore be read as specific to the breakpoints used in this study rather than as deployment-independent constants; we regard breakpoint recalibration as the single most consequential step required before any operational deployment. During the May 2026 deployment phase, the algorithm sporadically detected rare statistical co-occurrences. However, it could not by itself distinguish real-world chemical pollutants from instrumentation artifacts (e.g., a probe being out of water during sensor maintenance). The 2 km LoRa-based test at the SXCCE site was an excellent proof of concept. However, generalizability and pattern detection in other locations are limited. Future research will focus on extending the sensing network’s autonomy.

### 7.3. Future Work

We propose the following:

- **Edge Intelligence via TinyML:** The goal of this work is to deploy the T2MFRM solution for edge intelligence. TinyML will enable real-time, on-node pattern mining on ESP32-C3 gateways.
- **Automatic Fault Diagnosis:** Bearing in mind the findings of Section 6.4, it will be possible to integrate a secondary classifier, and, by doing so, it will also be possible to automatically distinguish sensor faults from real pollution events based on historical multivariate signs.
- **Expanded Sensor Fusion:** Future iterations of this work will include the detection of dissolved oxygen (DO) and ammonia (NH3) as part of the broader spectrum of industrial and organic pollutants.
- **Closed-Loop Feedback:** We will integrate the rare-pattern detection output with control valves, allowing the system to isolate a tank of polluted water when it detects a rare multivariate pattern.

## Figures and Tables

**Table 1:** Positioning of T2MFRM against the closest related methods.

Capability	FRI-Miner [11]	T2FM [20] (Prior Work)	T2MFRM (This Paper)
Rare pattern discovery	Yes	No (frequent only)	Yes
Type-2 fuzzy uncertainty	No (Type-1)	Yes	Yes
Validated on real field telemetry	No (synthetic)	No (synthetic)	Yes (Section 6)

**Table 2:** Summary of benchmark datasets used for evaluation.

Dataset	|D|	|I|	Avg. Tx Length	Max Tx Length	Type
Breast Cancer	699	10	10.0	10	Dense
Retail	88,162	16,470	10.3	76	Sparse
T10I4D100K	100,000	870	10.1	29	Sparse
Mushroom	8124	119	23.0	23	Dense
Chess	3196	75	37.0	37	Dense
Kosarak	990,002	41,270	8.1	2498	Sparse

**Table 3:** Multivariate rare itemsets identified by T2MFRM on real HydroSense AI field telemetry, ranked by support (N=644 valid transactions).

Itemset	Support S(X)	% of *N*	Member Transactions
Temp = High ∧ Turbidity = High	29.53	4.59%	100
pH = High ∧ Temp = High	9.53	1.48%	80
pH = High ∧ Turbidity = High	8.82	1.37%	80
pH = Low ∧ Temp = Low	8.76	1.36%	40
Temp = Low ∧ Turbidity = Low	7.89	1.23%	20

**Table 4:** Sensitivity of the flagship {pH = High AND Temp = High} rare pattern to breakpoint shift and FOU width (minRS=1%, minFS=15%). “No” means the pattern’s support fell outside the [minRS, minFS] band and was excluded from the rare-pattern output.

FOU Width	Breakpoint Shift	Flagged as Rare?	Support S(X)	% of *N*
Narrow (0.5%)	−10%	No	—	—
Narrow (0.5%)	−5%	No	—	—
Narrow (0.5%)	0% (baseline)	Yes	6.47	1.01%
Narrow (0.5%)	+5%	No	—	—
Narrow (0.5%)	+10%	No	—	—
Medium (1.0%)	−10%	No	—	—
Medium (1.0%)	−5%	No	—	—
Medium (1.0%)	0% (baseline)	Yes	9.71	1.52%
Medium (1.0%)	+5%	No	—	—
Medium (1.0%)	+10%	No	—	—
Wide (2.0%)	−10%	No	—	—
Wide (2.0%)	−5%	No	—	—
Wide (2.0%)	0% (baseline)	Yes	14.25	2.23%
Wide (2.0%)	+5%	No	—	—
Wide (2.0%)	+10%	No	—	—

**Table 5:** Joint minRS/minFS threshold sensitivity at baseline breakpoints, for the flagship {pH = High AND Temp = High} pattern.

FOU Width	minRS	minFS	#Rare Itemsets	Flagged as Rare?	Support S(X)
Narrow (0.5%)	1.0%	15.0%	6	Yes	6.47
Narrow (0.5%)	0.5%	10.0%	6	Yes	6.47
Narrow (0.5%)	2.0%	20.0%	7	No	—
Medium (1.0%)	1.0%	15.0%	6	Yes	9.71
Medium (1.0%)	0.5%	10.0%	6	Yes	9.71
Medium (1.0%)	2.0%	20.0%	7	No	—
Wide (2.0%)	1.0%	15.0%	7	Yes	14.25
Wide (2.0%)	0.5%	10.0%	6	Yes	14.25
Wide (2.0%)	2.0%	20.0%	9	Yes	14.25

**Table 6:** Computational efficiency across normal, fault-window, and drift-injected data conditions. Runtime and memory are mean ± st. dev. over 7 repeated runs on a single workstation.

Condition	*N*	#Itemsets	Runtime (ms)	Peak Mem (KB)	Throughput (tx/ms)
A (baseline)	638	6	189.4±21.2	271	3.4
B (normal-only)	613	4	167.6±5.4	257	3.7
C (fault window)	25	3	6.4±0.5	6	3.9
D (drift-injected)	638	7	177.7±5.6	272	3.6

A: Full clean deployment. B: Normal-only subset (real 8–9 May sensor-fault window excluded). C: That real fault window only (8–9 May, sensor artifact). D: Full deployment with additional synthetic electrochemical drift/noise injected.

## Data Availability

The field telemetry data supporting the findings reported in Section 6 are available from the corresponding author upon reasonable request. The MATLAB implementation and results underlying the sensitivity and robustness analysis in Section 6.6 (Table 4, Table 5 and Table 6), together with a prepared extract of the HydroSense AI telemetry used to generate them and a flow diagram of the analysis pipeline, are provided as Supplementary Materials with this submission and are permanently archived on Zenodo (DOI: https://zenodo.org/uploads/22767428). The six public benchmark datasets used for the Section 5 evaluation (Table 2) are available from the Frequent Itemset Mining Implementations (FIMI) repository, https://fimi.uantwerpen.be/data/, and the UCI Machine Learning Repository, https://archive.ics.uci.edu/datasets/.

## Supplementary Materials

The following supporting information is permanently archived on Zenodo and can be downloaded at https://zenodo.org/uploads/22767428: (i) T2MFRM_MATLAB_ Implementation.m, the fully documented MATLAB R2026a implementation used for the Section 6.6 sensitivity and robustness analysis (Table 4, Table 5 and Table 6); (ii) hydrosense_telemetry_clean.csv, the prepared HydroSense AI telemetry extract it reads; and (iii) a flow diagram of the analysis pipeline (provided as both PDF and PNG).
